# 27-Hydroxycholesterol Induces Aberrant Morphology and Synaptic Dysfunction in Hippocampal Neurons

**DOI:** 10.1093/cercor/bhy274

**Published:** 2018-11-03

**Authors:** Paula Merino-Serrais, Raul Loera-Valencia, Patricia Rodriguez-Rodriguez, Cristina Parrado-Fernandez, Muhammad A Ismail, Silvia Maioli, Eduardo Matute, Eva Maria Jimenez-Mateos, Ingemar Björkhem, Javier DeFelipe, Angel Cedazo-Minguez

**Affiliations:** 1 Division of Neurogeriatrics, Center for Alzheimer Research, Department of Neurobiology, Care Sciences and Society, Karolinska Institutet, Stockholm, Sweden; 2 Department of Neurology, Karolinska University Hospital Huddinge, Stockholm, Sweden; 3 Department of Physiology and Medical Physics Royal, College of Surgeons in Ireland, 123 St. Stephen's Green, Dublin 2, Ireland; 4 Division of Clinical Chemistry, Department of Laboratory Medicine, Karolinska University Hospital, Stockholm, Sweden; 5 Laboratorio Cajal de Circuitos Corticales (CTB), Universidad Politécnica de Madrid, Madrid, Spain; 6 Instituto Cajal, CSIC, Madrid, Spain; 7 Centro de Investigación Biomédica en Red sobre Enfermedades Neurodegenerativas (CIBERNED), ISCIII, Madrid, Spain

**Keywords:** 27-Hydroxycholesterol, Alzheimer’s disease, dendritic spines, dendritic arborization, pyramidal neuron, synaptic dysfunction

## Abstract

Hypercholesterolemia is a risk factor for neurodegenerative diseases, but how high blood cholesterol levels are linked to neurodegeneration is still unknown. Here, we show that an excess of the blood–brain barrier permeable cholesterol metabolite 27-hydroxycholesterol (27-OH) impairs neuronal morphology and reduces hippocampal spine density and the levels of the postsynaptic protein PSD95. Dendritic spines are the main postsynaptic elements of excitatory synapses and are crucial structures for memory and cognition. Furthermore, PSD95 has an essential function for synaptic maintenance and plasticity. PSD95 synthesis is controlled by the REST–miR124a–PTBP1 axis. Here, we report that high levels of 27-OH induce REST–miR124a–PTBP1 axis dysregulation in a possible RxRγ-dependent manner, suggesting that 27-OH reduces PSD95 levels through this mechanism. Our results reveal a possible molecular link between hypercholesterolemia and neurodegeneration. We discuss the possibility that reduction of 27-OH levels could be a useful strategy for preventing memory and cognitive decline in neurodegenerative disorders.

## Introduction

Cholesterol metabolism has been identified as a modulator of cognitive deficits associated with Alzheimer’s disease (AD) and other neurodegenerative disorders (Shobab et al. 2005; Hottman et al. 2014). Unlike cholesterol, its side-chain oxidized forms known as oxysterols, are able to traverse the blood brain barrier (BBB) from both directions (Björkhem et al. 2009). Higher levels of 27-hydroxycholesterol (27-OH) have been found in brains and cerebrospinal fluid (CSF) from AD patients (Heverin et al. 2004). Furthermore, we have shown that mice fed a cholesterol-enriched diet exhibit the same effect as high 27-OH levels: decreased levels of activity-regulated cytoskeleton-associated protein (Arc) (Mateos et al. 2009). In addition, high levels of 27-OH have been associated with memory deficits both in AD and other neurodegenerative processes through a liver X receptor(LXR)-dependent mechanism (Björkhem et al. 2009; Mateos et al. 2009; Mateos et al. 2011; Ismail et al. 2017). Moreover, 27-OH has been proposed as an endogenous selective estrogen receptor (ER) modulator (Brooks et al. 2017; Raza et al. 2017) and it has been proven that 27-OH induces the growth of ER-positive high-grade tumors (Nelson et al. 2011; Wu et al. 2013). A case–control study recently showed that increased plasma levels of 27-OH are associated with mild cognitive impairment in the elderly (Liu et al. 2016). Furthermore, it has been shown that 27-OH is a mediator of the negative effects of dietary cholesterol on cognition in mice (Heverin et al. 2015). However, the mechanisms by which hypercholesterolemia contributes to memory deficits and neurodegenerative disorders are not fully understood. Nowadays, the use of statins as therapeutic agents for dementia is extremely controversial with many doubting benefits (McGuinness et al. 2016). Some statins can cross the BBB, and therefore reduce the levels of cholesterol in the brain. Brain cholesterol levels are regulated in an autonomous manner due to the fact that plasma lipoproteins are prevented from crossing the BBB (Björkhem and Meaney 2004). It has been shown that cholesterol is an important player in the plasticity and synaptic function in the brain (Martin et al. 2014) as well as in the activation and AMPAR synaptic delivery during LTP (Brachet et al. 2015). Therefore, the reduction of brain cholesterol levels by statins could impair proper brain function. Additionally, there is an increasing conflict about the role of brain cholesterol level in AD (Ledesma and Dotti 2005; Gamba et al. 2012; Suzuki et al. 2013). Thus, the development of new and more selective targets is needed.

A large network of neuronal connections underlies the complex architecture of the central nervous system, and the correct development of neuronal connectivity is essential for proper brain function. In the cerebral cortex, dendritic spines (for simplicity, spines) constitute the major postsynaptic elements of glutamatergic synapses and are essential for memory, learning and cognition (Spruston 2008; DeFelipe 2015). Alterations on dendritic spines morphology have been described in relation with AD pathology and other neurodegenerative diseases (Knafo et al. 2009; Jimenez-Mateos et al. 2012; Merino-Serrais et al. 2013). The postsynaptic density (PSD) involves an extensive number of proteins and is essential for these processes (Grant and Husi 2001; Bayes et al. 2011). The PSD protein 95 (PSD95) is one of the most abundant proteins in the PSD and its function is considered critical for proper synaptic maturation and synaptic plasticity (El-Husseini et al. 2000; Schluter et al. 2006; Ehrlich et al. 2007; Frank and Grant 2017). PSD95, a scaffold protein almost exclusively located in the PSD (Hunt et al. 1996), assembles a specific set of signaling proteins on its PDZ domain including NMDA receptors, giving it an essential role in synaptic transmission, learning and memory (Cho et al. 1992; Hunt et al. 1996; Chen et al. 2005). The abundance of PSD95 is regulated at the RNA level by the polypyrimidine tract binding protein 1 (PTBP1) (Zheng et al. 2012), which is mainly expressed in non-neuronal and neuronal progenitor cells. After neuronal differentiation, PTBP1 levels are downregulated and the splicing pattern of the progenitor cells shifts towards a neuronal phenotype, including the generation of the mature isoform of PSD95 (Zheng et al. 2012). PTBP1 is not only a modulator during neuronal differentiation, but it is also an important regulator of lipid metabolism (Medina and Krauss 2013). The function of PTBP1 is regulated by a negative feedback loop by the REST complex, also called RE1 silencing transcription factor (NRSF). During embryonic development, REST is a transcriptional repressor of a large set of neuronal genes, leading the phenotypic transition of neural progenitors into mature neurons (Chong et al. 1995; Schoenherr and Anderson 1995; Ballas et al. 2005). Transcriptional repression of REST stimulates neuronal maturation and thus, REST has been proposed to be a link into the acquisition of a neuronal phenotype (Ballas et al. 2005). However, its function in neurodegeneration is still under discussion. REST is upregulated in human cortical and hippocampal neurons during normal aging and in response to neuronal insults, with a possible role against cell death, stress resistance and AD pathology (Zhao et al. 2017). It is also considered an antiapoptotic modulator in neuronal tumors (Fuller et al. 2005). Moreover, it has been shown that the overexpression of REST causes axon pathfinding errors leading to an aberrant neuronal morphology (Paquette et al. 2000). Among the genes repressed by REST there are several neuronal-specific microRNAs (miR), such as miR-124a and miR-9. MicroRNAs are considered key mediators in feedback regulatory networks, and their potential role on the pathogenesis behind neurodegenerative diseases has gained increasing attention (Johnston et al. 2005; Boutz et al. 2007; Eacker et al. 2009; Leung and Sharp 2013; Quinlan et al. 2017). MiR-124a was shown to inhibit REST synthesis through a complex autoregulatory loop during neuronal differentiation (Ballas et al. 2005; Conaco et al. 2006). MiR-124a also inhibits PTBP1, turning the REST-PTBP1 negative feedback loop into a positive one (Xue et al. 2013). Moreover, it has been shown that miR-124a regulates neurite outgrowth and elongation by targeting oxysterol-binding protein (OSBP) (Gu et al. 2016). MiR-9 is one of the most abundant miRNAs in the brain, and has been shown to be a potent inducer of dendritic growth through the downregulation of REST (Lagos-Quintana et al. 2002; Krichevsky et al. 2003; Giusti et al. 2014).

In view of the relationship between hypercholesterolemia and high levels of 27-OH, we hypothesized that 27-OH could be a molecular link between hypercholesterolemia and cognitive decline. In the present study, we explore the impact of high 27-OH levels on hippocampal neuronal morphology, PSD95 levels and a possible role of the REST–miR124a–PTBP1 axis in these effects. Therefore, affecting neurotransmission and leading to impairment in memory and cognition. Increased knowledge about regulation of the miRNAs levels could offer us an alternative target for drugs discovery programs.

Using both in vivo and in vitro models, we found that high levels of 27-OH diminish dendritic arborization, spine density and PSD95 levels in the hippocampus. Our results point out that decreased PSD95 levels are caused by a REST–miR124a–PTBP1 axis dysregulation and suggest a crucial role for the nuclear retinoic receptor X gamma (RXRγ) in the dysregulation of the system. These findings reveal a novel pathological mechanism that links high blood cholesterol levels with memory impairment, and identify possible alternative targets for drugs discovery programs.

## Material and Methods

### Isolation and Culture of Hippocampal Rat Primary Neurons

Hippocampal primary neuronal cultures from E18 Sprague-Dawley rat embryos were established as previously reported (Mateos et al. 2009). Neurons at 10 days in vitro (DIV) were treated from 1 DIV with 27-OH (final concentration 1 μM, Steraloids; Newport, RI, USA), the LXR agonists GW3965 (final concentration 1 μM, Sigma) and TO-901317 (final concentration 1 μM, Sigma), β-estradiol (final concentration 10 nM Sigma), the ER antagonist ICI (final concentration 100 nM, Abcam), once by incubation every day. Additionally, hippocampal primary neurons were treated with 27-OH (1 μM) from 1 to 4 DIV and then allowed to grow until 10 DIV (Treatment II). For the time course experiment, the hippocampal primary neurons were treated daily with 27-OH (1 μM) and then collected at different time points: 1 (previous treatment), 2 and 4 DIV. For the control group, the neurons were treated with an equal amount of vehicle (97% ethanol). Experiments with primary cultures were conducted with approval from the regional ethical committee of Karolinska Institutet.

### Immunocytochemistry

Immunofluorescence was performed as previously reported (Ismail et al. 2017) on hippocampal primary neurons after administration of the treatments indicated above. Single and double immunofluorescence was performed with the following primary antibodies: chicken anti-MAP2 (1:10 000 dilution, Abcam, ab5392) and mouse anti-PSD95 (1:1000 dilution, Abcam, ab2723). Cells were then incubated for 1 h at room temperature at a dilution of 1:1000 with secondary antibodies; Alexa fluor 633 goat antichicken and Alexa flour 488 goat antimouse (Invitrogen, A11035 and A11055, respectively). Identification of the nuclei of cell bodies was performed using 4, 6 diamino-2-phenylindole (DAPI; Sigma) and the F-actin was stained by Alexa Fluor 488-Phalloidin (1:1000; Life Technologies). Omission of the primary antibody was done as a control staining. Coverslips were finally mounted onto glass slides using fluorescence mounting medium (DAKO Cytomation, Glostrup, Denmark). Imaging was performed with a Zeiss (LSM 510 META) confocal laser scanning system using a 488 nm Argon laser, 633 nm Helium–neon laser and UV 405 nm. The fluorescence of DAPI, Alexa 488 and Alexa 633 was recorded through separate channels.

### Apoptosis Assay

Irreversible DNA damage in adherent cell was detected using a fluorescein-based TUNEL kit (Promega), according to the manufacturer’s instructions. To test the reliability of the assay a positive and negative control were performed.

### Cell Reconstruction

Neurons were reconstructed in 3D using Neurolucida software (MicroBrightField Inc., Williston, VT). The morphological parameters were determined by tracing the images of the acquired dendritic trees. After tracing, the reconstructed data were exported to Neurolucida Explorer (MicroBrightField Inc., Williston, VT) for quantitative analysis. Several morphological parameters were measured to assess the complexity of the dendritic arbor: total dendritic length, total number of intersections, total number of branches and somatic area. For further analysis, dendritic length and number of intersections were also analyzed as a function of the distance from the soma, creating concentric spheres centered on the cell body of increasing 10-μm radii (Sholl analysis). Soma areas were measured by drawing soma contours. To assure impartiality all morphological analysis was performed blindly by the same investigator.

### RNA Extraction, Real Time RT-qPCR and miRNA Expression

Total RNA extraction, reverse transcription and real-time quantitative PCR (RT-qPCR) amplification assays for PSD95, REST and PTBP1 were performed using specific primers (Life Technologies) as previously reported (Tajeddinn et al. 2016). mRNA copy numbers of all target transcripts were adjusted by mRNA copy numbers of GAPDH and the values calculated were compared with controls (set at 100%). Expression of miR-124a and miR-9 was measured in the whole hippocampus of Cyp27Tg and WT mice using stem-loop specific primers for mmu-miR-124a or mmu-miR-9 (Life Technologies) as previously reported (Jimenez-Mateos et al. 2015). Expression of RNU19 was used for normalization. A relative fold change in expression of miR-124a and miR-9 was determined using the 2^−∆∆CT^ method.

### Immunoblotting Analyses

Western blot analysis was carried out as aforementioned (Mateos et al. 2009). Following the transfer to a nitrocellulose membrane (Schleicher & Schuell, Germany), milk blocked blots were incubated with the following antibodies: mouse anti-PSD95 (1:1000; Abcam), rabbit anti-REST (1:1000; Millipore), rabbit anti-PTBP1 (1:1000; Bethyl Laboratories), mouse antipan SHANK (1:1000; Millipore), mouse anti-SNAP25 (1:1000; Transduction laboratories), rabbit anti-NeuN (1:500; Millipore) and rabbit anti-GAPDH (1:1000; Enzo) overnight. Secondary incubation was done using antirabbit or antimouse IgG at a 1:5000 dilution at room temperature (Amersham Biosciences, Little Chalfont, England). Immunoreactivity was detected by ECL detection system (Amersham, Biosciences, Little Chalfont, England). The densitometry analyses of the immunoreactive bands were performed using ImageJ software (NIH, MA, USA). Some immunoblots were stripped using Restore™ Western Blot Stripping buffer (Pierce, Rockford, IL, USA) at room temperature for 15 min and then blocked and re-blotted with other antibodies.

### Silencing of Retinoic Acid Receptor Gamma (RXRγ)

To knockdown endogenous RXRγ, we used a commercially available siRNA pool directed towards different coding regions of the RXRγ rat gene (L-083061-02, ON-TARGETplus siRNA SMARTpool, Dharmacon Inc.). After 1 days in vitro (DIV), hippocampal primary neurons (80% confluence) were treated with either 50 nM of RXRγ siRNA or nontargeting siRNA (SCR) for 12 h. The media was thereafter removed and replaced with fresh complete neurobasal media plus 1 μM 27-OH or equivalent 97% ethanol for controls. The treatments were kept for 24 h and the cells were harvested in cold as described previously. Knockdown efficiency was monitored by SDS-PAGE western blot followed by densitometry analysis.

### Animals

#### Cyp27Tg

Generation and breeding of the enzyme sterol 27-hydroxylase (Cyp27A1) overexpressing mice (Cyp27Tg) has been described previously (Meir et al. 2002; Bavner et al. 2010; Ismail et al. 2017). Cyp27A1 is responsible for converting cholesterol to 27-OH (Bavner et al. 2010). Cyp27Tg mice have 5–6 times higher levels of 27-OH than control mice (WT) in serum (283 ± 11 vs. 48 ± 2 ng/mL) and in brain (3.5 ± 0.5 vs. 0.3 ± 0.0 ng/mg) (Ismail et al. 2017). Cyp27Tg mice have normal brain cholesterol levels but a modest upregulation of cholesterol synthesis (Ali et al. 2013). Age-matched littermates without the transgene (WT) were used as controls. C57BL/6 J is the background mouse strain of all mice used in the study. The mice were fed normal chow and water provided ad libitum. The animals were housed in groups of 4–5 with 12 h light/dark cycle. Only 7–8-week-old mice (males) were used. For mRNA expression and immunoblotting analyses the animals were sacrificed by decapitation, the brains were dissected and immediately frozen on dry ice and stored at –80 °C until further analysis. Different animals were used to perform mRNA expression and immunoblotting analysis. A complete right hippocampus per animal was included in the analysis. For intracellular injections and immunohistochemistry the animals were overdosed by intraperitoneal injection of sodium pentobarbitone and perfused intracardially with 4% paraformaldehyde (PFA). Experimental procedures were conducted in accordance with the European regulation and approved by the ethical committee of Karolinska Institutet.

### Intracellular Injections

Mice were overdosed by intraperitoneal injection of sodium pentobarbitone and perfused intracardially with 4% PFA. Their brains were postfixed in 4% PFA for 24 h. Coronal sections (150μm) from the right hemisphere were obtained on a vibratome. Sections were prelabeled with DAPI. Pyramidal neurons located in the middle of the pyramidal cell layer of the CA1 region were then individually injected with Lucifer Yellow (LY) by continuous current. Neurons were injected until the distal tips of each dendrite fluoresced brightly, indicating that the dendrites were completely filled and ensuring that fluorescence did not diminish at a distance from the soma. Following intracellular injections, the sections were processed with a rabbit antibody against LY (produced at the Cajal Institute) (1:400 000 in stock solution: 2% BSA [A3425; Sigma], 1% Triton X-100 [30 632; BDH Chemicals], 5% sucrose in phosphate buffer [PB]) and antibody binding was then detected with a biotinylated donkey antirabbit secondary antibody (1:200, RPN1004, Amersham Pharmacia Biotech), followed by an incubation with streptavidin coupled to Alexa fluor 488 (1:1000 in PB, Molecular Probes, Eugene, OR, USA). Finally, the sections were washed in PB and mounted with ProLong Gold Antifade Reagent (Invitrogen Corporation, Carlsbad, CA, USA). Imaging was performed with a Nikon Ti-E inverted point scanning confocal system using a 488 nm Argon laser and UV 405 nm. The fluorescence of DAPI and Alexa 488 was recorded through separate channels.

### Cell Reconstruction and Spine Density Analysis in Cyp27Tg

The complexity of the basal and apical arborization from LY-injected neurons was determined in 3D with Neurolucida software (described above). For spine density analysis, spines were marked with Neurolucida during tracing and all protrusions were considered as spines, applying no correction factors to the spine counts. Spine density was calculated for each dendrite by dividing the number of spines by dendritic length using Neurolucida Explorer. In addition, spine density was measured as a function of the distance from the soma (Sholl analysis). Subsequently, the length of the dendritic segment was divided by the number of spines in each 10 μm stretch from the origin. To assure impartiality all morphological analysis was performed blindly by the same investigator.

### Immunohistochemistry

Mice were overdosed by intraperitoneal injection of sodium pentobarbitone and perfused intracardially with 4% PFA. Their brains were postfixed in 4% PFA for 24 h. Coronal sections (20μm) from the left hemisphere were obtained on a cryostat. Free-floating sections were blocked for 1 h in PB with 0.25% Triton-X and 3% normal goat (Vector Laboratories Inc., Burlingame, CA, USA). Double immunofluorescence was performed with the primary antibodies: mouse anti-PSD95 (1:500; Abcam, ab2723) and rabbit anti-NeuN (1:500; Millipore). Sections were then incubated for 2 h at room temperature at a dilution of 1:1000 with the secondary antibody Alexa fluor 633 goat antimouse (Invitrogen, A11035) and Alexa fluor 488 goat antirabbit (Invitrogen, A1141875). Omission of the primary antibody was done as a control staining. Sections were finally mounted using fluorescence mounting medium (DAKO Cytomation, Glostrup, Denmark). Imaging was performed with a Nikon Ti-E inverted point scanning confocal system using a 640 diode and Argon lasers. The fluorescence of Alexa 488 and Alexa 633 was recorded through separate channels. Fluorescence intensity was measured with the ImageJ 1.383 software (NIH, MA, USA). Three sections per animal were used for the analysis. To assure impartiality the analysis was performed blindly by the same investigator.

### Stereotaxic Injections

The 7–8-week-old C57BL/6 male mice were anesthetized with isofluorane/O2 and placed on a stereotaxic frame (David Kopf Instruments) on a heated pad at 37 °C to maintain a normal body temperature. Injections were done bilaterally into the lateral ventricle with 1 μL of solution per side using a 10 μL gauge Hamilton syringe. A 27-OH (Steraloids; Newport, RI, USA) was associated with HDL protein (Abcam, ab77881) at a concentration ratio of 1:3 and incubated together at 37 °C for 1 h and diluted in artificial CSF (aCSF; RD Systems, 3525) and used in a final concentration of 5 μM (27CSF). The control group was injected under the same conditions with aCSF. The stereotaxic coordinates for the injection site in the lateral ventricle were −0.9 mm anteriorposterior, ± 1.4 mm lateral to bregma and 2.0 mm from the skull surface according to the Paxinos Mouse brain atlas (Franklin and Paxinos 2001).

Experimental procedures were conducted in accordance with the European regulation and approved by the ethical committee of Karolinska Institutet.

### Statistical Analysis

For all the morphological parameters measured, the values were averaged per neuron to produce a mean per group. Each group originates from a separate animal. To test the overall effect, a Mann–Whitney test was used to compare the averages. When more than 2 groups were compared, a one-way ANOVA was used, followed by Bonferroni multiple comparison post-hoc test. Two-way ANOVA repeated measures (*P* and *F* values + interaction are shown) followed by a post-hoc multiple Bonferroni test was used to compare values when presented as a function of the distance from the soma.

For Immunoblotting and RT-qPCR analyses a Mann–Whitney test was used to compare the averages. When more than 2 groups were compared (siRNA measurements), one-way ANOVA was used followed by Bonferroni multiple comparison post-hoc test. Data values are expressed as mean ± standard error (SEM). In all cases, *P* < 0.05 was considered to be significant (* < 0.05, ** < 0.01, *** < 0.001).

## Results

### 27-OH Treatment Diminishes Dendritic Arborization, Spine Density and PSD95 Levels

We first evaluated DNA damage in hippocampal primary neurons treated with 27-OH (1 μM) every day from 1 to 10 days in vitro (DIV) by performing a TUNEL assay. We found similar staining in control neurons and neurons treated with 27-OH (Supplementary Fig. S1A, B).

**Figure 1.**
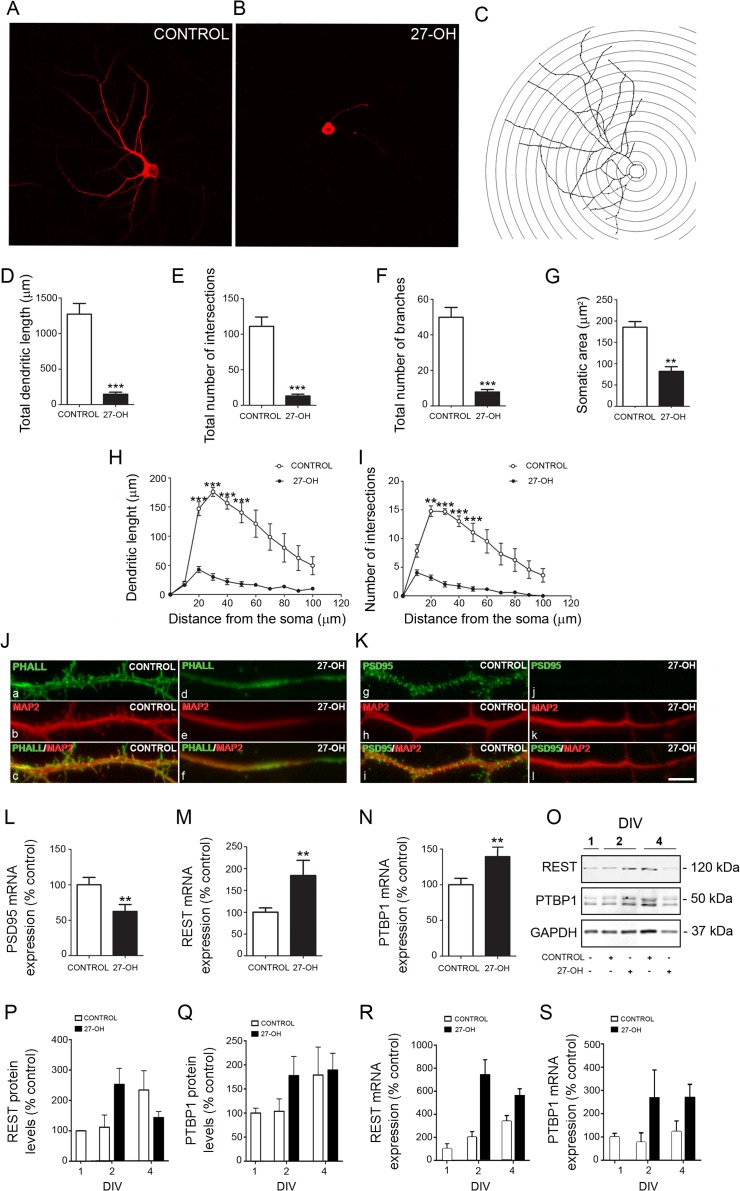
27-OH treatment modulates dendritic arborization, spine density, PSD95 synthesis and the REST–PTBP1–PSD95 axis in vitro. (*A*, *B*) Confocal images showing the morphological structure of (*A*) control neurons and (*B*) neurons treated with 27-OH stained with the somatodendritic marker MAP2. (*C*) Representative picture of the Sholl analysis of the same neuron shown in *A*. (*D*–*I*) Comparative morphometric analysis of (*D*, *H*) dendritic length, (*E*, *I*) number of intersections, (*F*) number of branches and (*G*) somatic area for control neurons and neurons treated with 27-OH (*n* = 6). Data presented as (*D*–*G*) the average per neuron and (*H*, *I*) as a function of distance from the soma (*n* = 6). (*J*, *K*) Representative images of dendrites from control neurons (a–c; g–i) and neurons treated with 27-OH (d–f; j–l) stained using phalloidin (PHALL) (a,c,d,f), anti-MAP2 (b,c,e,f,h,i,k,l) and anti-PSD95 (g,i,j,l). Neurons treated with 27-OH showed less dendritic spines and PSD95 compared with control neurons. Scale bar (in *l*) *A*–*C*: 30 μm; *J*, *K*: 10 μm. (*D*–*G*) Mann–Whitney test was used to compare averages, (*H*, *I*) two-way ANOVA followed by a post-hoc multiple Bonferroni comparison was used in the Sholl analysis. (*L*–*N*) 27-OH treatment modulates PSD95, REST and PTBP1 expression levels in vitro. (*L*) PSD95, (*M*) REST and (*N*) PTBP1 mRNA expression levels in hippocampal primary neurons treated with 27-OH (1 μM) (*n* = 12) compared with control neurons (*n* = 15). (*P*, *Q*) Time course analysis showing REST and PTBP1 protein (*P*, *Q* respectively) and mRNA levels (*R*, *S* respectively) at different time points (1, 2 and 4 DIV). All data are represented as mean ± SEM; **P < 0.01, ***P < 0.001.

Next, we evaluated the effect of high 27-OH levels on dendritic arborization, spine density and PSD95 levels. Hippocampal primary neurons were treated every day with 27-OH (1 μM) and compared with control (vehicle treated) neurons. In this study, we reconstructed in 3D the entire dendritic tree of 70 hippocampal primary neurons (35 from control group and 35 from the group treated with 27-OH; Fig. 1) by confocal microscopy (40×/1.3 Oil). Each image stack (image size = *x*: 224.78 μm, *y*: 224.78 μm, *z*: 1.00 μm) contained a full simple neuron. We evaluated the dendritic arborization of individual neurons using the somatodendritic marker MAP2 (Fig. 1*A*,*B*) and considering as morphometric parameters: dendritic length, number of intersections, number of branches and somatic area. Neurons treated with 27-OH showed a widespread pathological morphology with extremely short neurites compared with control. Further analysis showed that 27-OH treatment significantly reduced total dendritic length (control = 1271 ± 150.8; 27-OH = 145 ± 29.28; *P* = 0.002; *n* = 6; Mann-Whitney; Fig. 1*D*), total number of intersections (control = 111 ± 12.98; 27-OH = 13.22 ± 2.51; *P* = 0.002, *n* = 6; Mann-Whitney; Fig. 1*E*), total number of branches (control = 49.89 ± 5.5; 27-OH = 7.84 ± 1.39; *P* = 0.002; *n* = 6; Mann-Whitney; Fig. 1*F*) and somatic area (control = 185.5 ± 13.37; 27-OH = 82.11 ± 10.77; *P* = 0.002; *n* = 6; Mann-Whitney; Fig. 1*G*). Moreover, we found that 27-OH induces a significant reduction of dendritic length (Sholl analysis; two-way ANOVA, *P* < 0.0001; *F* = 36.57; Bonferroni post-hoc test, 20, 30, 40, 50 μm, *P* < 0.001; Fig. 1*H*) and number of intersections analyzed as a function of the distance from the soma (Sholl analysis; two-way ANOVA, *P* < 0.0001; *F* = 23.48; Bonferroni post-hoc test, 20 μm, *P* < 0.01; 30, 40, 50 μm, *P* < 0.001; Fig. 1*I*).

We next analyzed the effect of 27-OH treatment on dendritic spine density and PSD95 levels by confocal microscopy using Phalloidin staining and PSD95 immunostaining (Fig. 1J and 1K, respectively). As seen in Figure 1*J*, neurons treated with 27-OH showed an extreme reduction in the number of spines and PSD95 immunostaining signal (Fig. 1*K*) compared with control neurons.

### 27-OH Treatment Modulates PSD95, REST and PTBP1 Levels

Considering the relevance of the REST–miR124a–PTBP1 axis on neuronal differentiation, we next determined whether 27-OH affects the mRNA expression of PSD95, REST and PTBP1. RT-qPCR analysis showed significantly lower PSD95 expression (control = 100 ± 10.47; 27-OH = 62.52 ± 9.69; Mann-Whitney, *P* = 0.007; Fig 1*L*) and increased expression of REST (control = 100 ± 9.92; 27-OH = 184.2 ± 34.67; Mann-Whitney, *P* = 0.007; Fig. 1*M*) and PTBP1 (control = 100 ± 9.06; 27-OH = 139.5 ± 12.37; Mann-Whitney, *P* = 0.004, Fig. 1*N*) in hippocampal primary neurons treated with 27-OH (*n* = 10) (1 μM, 1–10 DIV) compared with control (*n* = 15).

To further study the effect of 27-OH on neuronal morphology during differentiation we treated neurons with 27-OH (1 μM) and collected samples for western blot and RT-qPCR analysis at different days in vitro (DIV). As observed in Figure 1, 27-OH lead to a 229% increase in REST (control = 110 ± 40; 27-OH = 252 ± 52; *n* = 3; Fig. 1*O*,*P*) and 75% increase in PTBP1 (control = 103.6 ± 25.79; 27-OH = 178 ± 39; *n* = 3, Fig. 1*O*,*Q*) protein levels compared with control after 2 DIV. This increase in protein levels was accompanied by increased mRNA expression of both proteins: 350% increase in REST (control = 200 ± 80; 27-OH = 740 ± 225; *n* = 3; Fig. 1*R*) and 380% increase in PTBP1 (control = 76 ± 70; 27-OH = 266 ± 200; *n* = 3; Fig. 1*S*). At 4 DIV, PTBP1 protein levels in 27-OH treated neurons were similar to control (control = 179 ± 58; 27-OH = 189 ± 34; *n* = 3; Fig. 1*Q*) while REST protein levels decreased in 27-OH treated neurons (control = 233 ± 63; 27-OH = 142 ± 57; *n* = 3; Fig. 1*P*). In the case of mRNA expression levels a 165% increase was found in REST (control = 338 ± 83; 27-OH = 558 ± 107; *n* = 3; Fig. 1*R*) and a 220% increase in PTBP1 (control = 122 ± 77; 27-OH = 269 ± 97; *n* = 3; Fig. 1*S*).

In order to test whether early effects in the REST–miR124a–PTBP1 axis are sufficient to trigger the morphological alterations previously observed (Fig. 1), we treated neurons with the same concentration of 27-OH (1 μM) but just from 1 to 4 DIV, allowing them to differentiate up to 10 DIV (Treatment II). Determination of dendritic arborization, spine density and PSD95 levels showed that this treatment led to the same alterations as in neurons treated from 1–10 DIV (Supplementary Fig. S1). Analysis of PSD95 mRNA expression showed decreased PSD95 expression in neurons treated with 27-OH (1 μM; 1–4 DIV) compared with control (control = 100 ± 10.47; 27-OH = 65.16 ± 4.58; *n* = 12). One-way ANOVA, *P* = 0.007; Supplementary Fig. S1).

### LXR and ER Synthetic Ligands do not Alter Dendritic Arborization and PSD95 Levels

**Figure 2.**
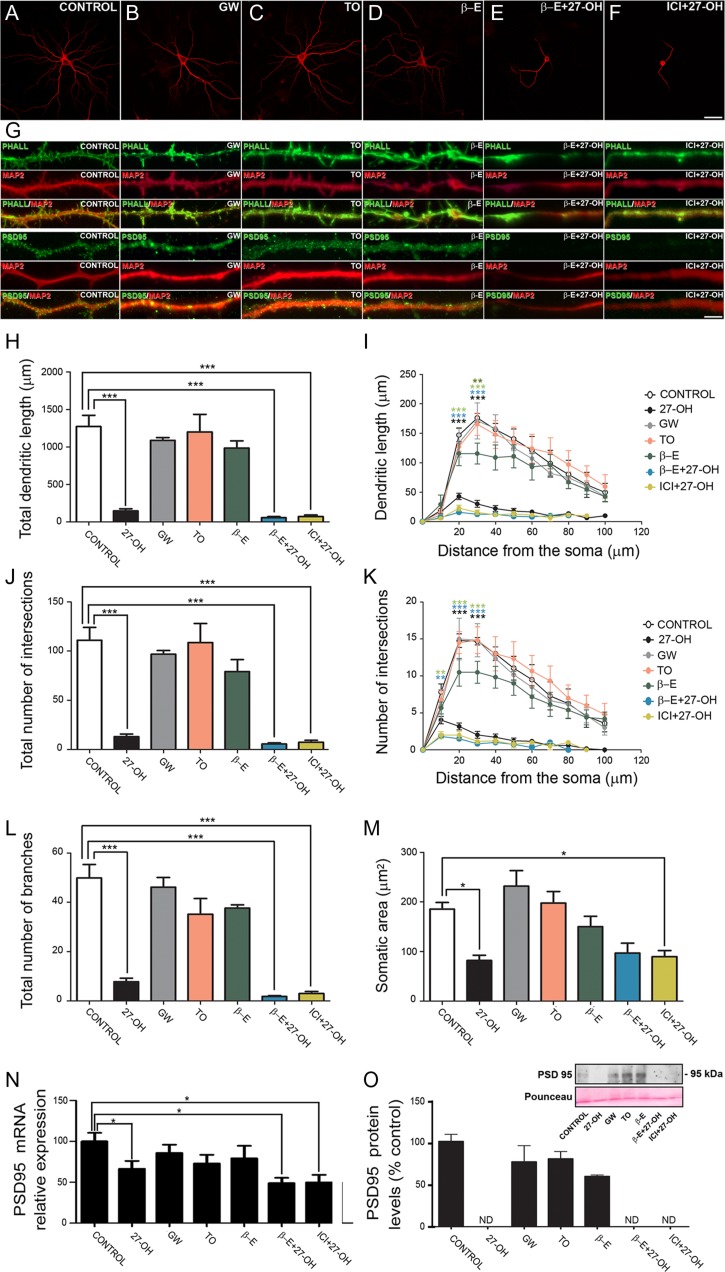
Role of LXR and ER on 27-OH treatment in vitro. (*A*–*F*) Confocal images showing the dendritic arborization using the somatodendritic marker MAP2 of (*A*) control neurons, neurons treated with the LXR ligands: (*B*) GW3965 (GW, 1 μM) and (C) TO-901317 (TO, 1 μM), (*D*) β-Estradiol (β-E; 10 nM), (*E*) β-Estradiol +27-OH (β-E+27-OH; 10 nM and 1 μM, respectively) and (*F*) the ERs antagonist + 27-OH (ICI+27-OH; 100 nM and 1 μM, respectively). (*G*) Representative images of dendrites from control neurons and neurons treated with GW, TO, β-E, β-E+27-OH and β-E+ICI stained using phalloidin (PHALL), anti-MAP2 and anti-PSD95. Higher magnification in Supplementary Fig. S2. Scale bar (in *F*): *A*–*F*: 30 μm; *G*: 10 μm. (*H*–*M*) Comparative morphometric analysis of (*H*, *I*) dendritic length, (*J*, *K*) number of intersections, (*L*) number of branches and (*M*) somatic area for control neurons and neurons treated with 27-OH, GW, TO, β-E, β-E+27-OH and ICI+27-OH (*n* = 6). Data presented as (*H*, *J*, *L*, *M*) the average per neuron and (*I*, *K*) as a function of distance from the soma. (*H*, *J*, *L*, *M*) One-way ANOVA followed by a post-hoc multiple Bonferroni comparison was used to compare averages, (*I*, *K*) two-way ANOVA followed by a post-hoc multiple Bonferroni comparison was used in the sholl analysis. (*N*) Real time RT-qPCR analysis of PSD95 mRNA levels in the same groups described above (*n* = 4–15). One-way ANOVA followed by Mann-Whitney test was employed. (*O*) Western blot and densitometry analysis showing the protein level of PSD95 (normalized to GAPDH) in control neurons and neurons treated with 27-OH, GW, TO, β-E, β-E+27-OH and ICI+27-OH (*n* = 3). In neurons treated with 27-OH, β-E+27-OH, and ICI+27-OH, the signal of PSD95 was not detectable (ND). All data are represented as mean ± SEM; *P < 0.05, **P < 0.01, ***P < 0.001.

As 27-OH is an endogenous ligand of LXR, we next analyzed if the effects observed in 27-OH treated neurons were mediated by LXR activation. Hippocampal primary neurons were treated with 2 different LXR ligands: GW3965 (GW, 1 μM) and TO-901317 (TO, 1 μM) at concentrations previously shown to activate LXR (Mateos et al. 2011; Do et al. 2016). Additionally, since 27-OH is an endogenous selective ER modulator (Brooks et al. 2017; Raza et al. 2017) we explored whether 27-OH effects on dendritic arborization were mediated through ERs. We treated neurons with β-Estradiol (β-E, 10 nM) and the ER antagonist (ICI, 100 nM) at concentrations shown to activate ERs (Liu et al. 2013; Zhao et al. 2016). We evaluated the dendritic arborization of individual neurons under the treatments described above, using the somatodendritic marker MAP2 (Fig. 2*A*–*M*; Supplementary Fig. S2). The morphological parameters measured were: total dendritic length (control = 1271 ± 150.8; 27-OH = 145 ± 29.28; GW = 1087 ± 38.57; TO = 1200 ± 233.7; β-E = 986.5 ± 94.96; β-E + 27-OH = 57.80 ± 12.62; ICI + 27-OH = 72.18 ± 20.69; *n* = 6; one-way ANOVA, *P* < 0.001; Bonferroni post-hoc test, *P* < 0.001; Fig. 2*H*), dendritic length as a function of the distance from the soma (Sholl analysis; two-way ANOVA, *P* < 0.0001; *F* = 12.41; Bonferroni post-hoc test, 20, 30 μm, *P* < 0.001; Fig. 2*I*), total number of intersections (control = 111 ± 12.98; 27-OH = 13.22 ± 2.51; GW = 96.83 ± 3.85; TO = 108 ± 19.28; β-E = 79.17 ± 12.16; β-E + 27-OH = 5.66 ± 1.14; ICI + 27-OH = 7.33 ± 2.17; *n* = 6; one-way ANOVA, *P* < 0.001; Bonferroni post-hoc test, *P* < 0.001; Fig. 2*J*), number of intersections as a function of the distance from the soma (Sholl analysis; two-way ANOVA, *P* < 0.001; *F* = 8.9; Bonferroni post-hoc test, 10 μm, *P* < 0.01; 20, 30 μm, *P* < 0.001; Fig. 2*K*), total number of branches (control = 49.89 ± 5.5; 27-OH = 7.84 ± 1.39; GW = 46.17 ± 3.92; TO = 35.17 ± 6.34; *n* = 6; β-E = 37.67 ± 1.3; β-E+ 27-OH = 1.83 ± 0.30; ICI + 27-OH = 3 ± 0.81; one-way ANOVA, *P* < 0.001; Bonferroni post-hoc test, *P* < 0.001; Fig. 2*L*) and somatic area (control = 185.5 ± 13.37; 27-OH = 82.11 ± 10.77; GW = 232.2 ± 31.15; TO = 197.8 ± 23.27; β-E = 150.6 ± 20.85; β-E+ 27-OH = 97.03 ± 19.85; ICI + 27-OH = 89.99 ± 11.92; *n* = 6; one-way ANOVA, *P* < 0.001; Bonferroni post-hoc test, *P* < 0.05; Fig. 2*M*).

We next analyzed the effect of LXR and ER synthetic ligands treatment on dendritic spine density and PSD95 levels by confocal microscopy using Phalloidin staining and PSD95 immunostaining (Fig. 2*G*; Supplementary Fig. S2). As seen in Figure 2*A*–*M*, no changes in dendritic arborization, spine density and PSD95 signal were found in neurons treated with LXR ligands compared with controls. However, we found a significant decrease in the groups treated with 27-OH, β-E+ 27-OH and ICI + 27-OH compared with control neurons in all the parameters analyzed.

Additionally, RT-qPCR analysis showed a significant reduction in PSD95 mRNA levels in the groups treated with 27-OH, β-E + 27-OH and ICI + 27-OH compared with control (control = 100 ± 10.47; 27-OH = 66.46 ± 9.6; GW = 85.81 ± 10.09; TO = 72.96 ± 10.61; β-E = 79.34 ± 15.24; β-E+ 27-OH = 48.99 ± 6.39; ICI + 27-OH = 49.84 ± 9.23; *n* = 3–15; one-way ANOVA, *P* < 0.05; Mann-Whitney, *P* = 0.01, *P* = 0.023, *P* = 0.037, respectively; Fig. 2*N*). Moreover, no differences were found in PSD95 protein levels in neurons treated with LXR ligands (GW = 76.22 ± 18.86; TO = 79.77 ± 8.42; *n* = 3) and β-E (59.08 ± 1.57; *n* = 3) when we compared with control neurons (100 ± 1.57; *n* = 3; one-way ANOVA, *P* > 0.05; Fig. 2*O*). No detectable signal was found in neurons treated with 27-OH, β-E + 27-OH, and ICI + 27-OH (Fig. 2*O*).

### RXRγ as a Possible Mediator in the Effects of 27-OH Treatment

**Figure 3.**
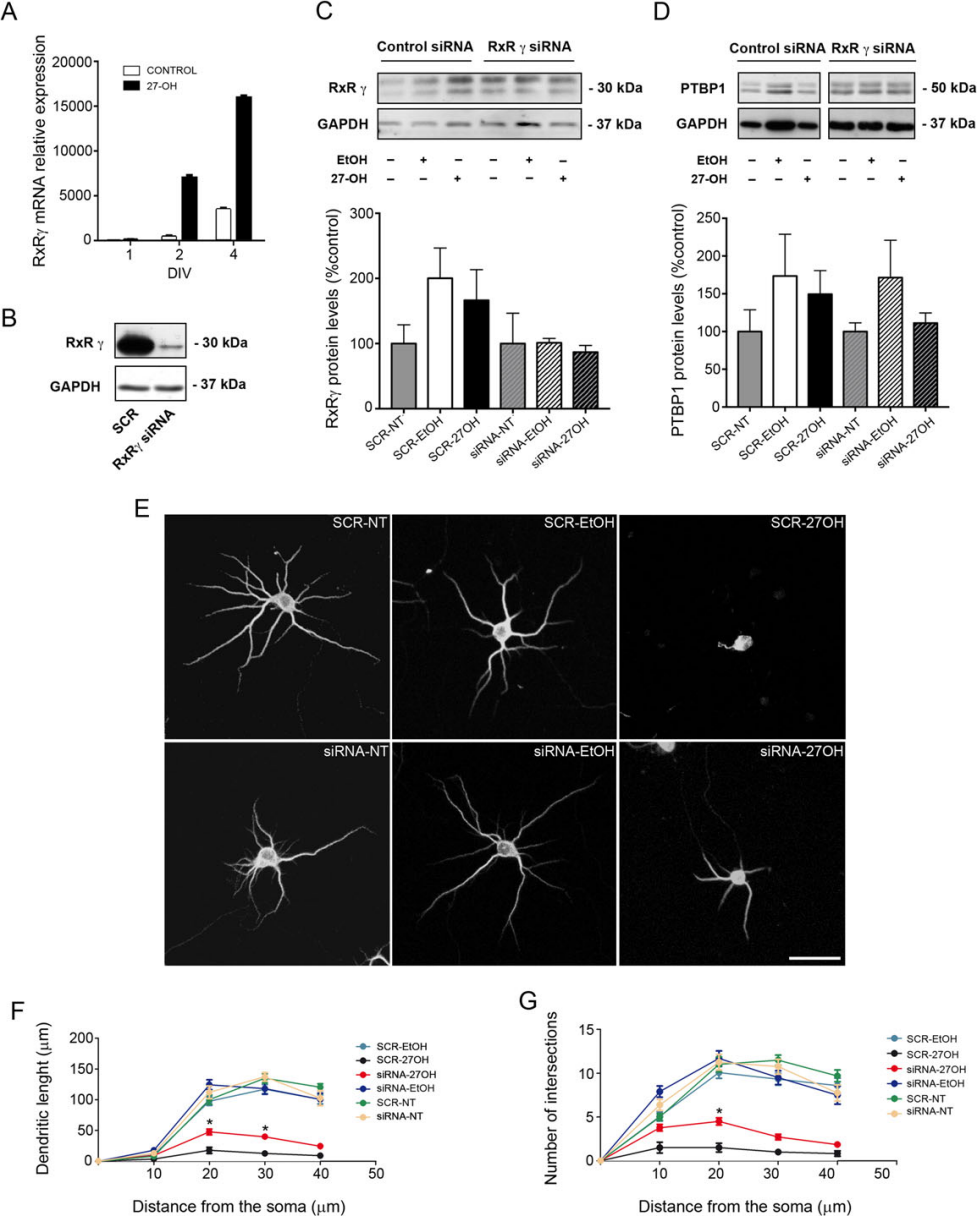
Role of RxRγ on 27-OH treatment in vitro. (*A*) Time course analysis showing RxRγ mRNA levels at different time points (1, 2 and 4 DIV). (*B*) Western blot of RxRγ 72 h after transfection in nontargeting siRNA (SCR) and RxRγ siRNA transfected neurons (RxRγ siRNA). (*C*, *D*) Western blot of RxRγ and PTBP1 and densiometry analysis. The groups included in the analysis were: neurons transfected with nontargeting siRNA with no treatment (SCR-NT), transfected with nontargeting siRNA and treated with EtOH (SCR-EtOH), transfected with nontargeting siRNA and treated with 27-OH (SCR-27OH), transfected with RxRγ siRNA and no treatment (siRNA-NT), transfected with RxRγ siRNA and treated with EtOH (siRNA-EtOH) and transfected with RxRγ siRNA and treated with 27-OH (siRNA-27OH). One-way ANOVA (*P* > 0.05) was employed. (*E*) Confocal images showing the neuronal structure of hippocampal primary neurons stained with the somatodendritic marker MAP2. Scale bar: 30 μm. (*F*, *G*) Comparative morphometric analysis of (*F*) dendritic length and (*G*) number of intersections as a function of distance from the soma. Two-way ANOVA followed by a post-hoc multiple Bonferroni comparison was used in the analysis. All data are represented as mean ± SEM; *P < 0.05.

Retinoid X receptors (RxR) have been described as very important modulators in the maintenance of lipid homeostasis (Shulman and Mangelsdorf 2005; Sentinelli et al. 2013), brain development, dendritic outgrowth, memory and cognition (Dolle et al. 1994; Chiang et al. 1998; Calderon and Kim 2007; Mingaud et al. 2008). For this reason, we next aimed to test whether the effect of 27-OH over the REST–miR124a–PTBP1 axis could be RXRγ-mediated. We treated neurons with 27-OH (1 μM) and collected samples for RT-qPCR analysis at different days in vitro (DIV). As shown in Figure 3*A*, RxRγ mRNA expression levels increased after 2 DIV (control = 433 ± 125; 27-OH = 7000 ± 300; *n* = 3) and 4 DIV (control = 4347 ± 164; 27-OH = 15957 ± 215; *n* = 3; Fig. 3*A*).

To clarify whether the effect of 27-OH could be mediated by RxRγ, we transfected neurons with a siRNA that efficiently decreased RxRγ protein levels 72 h after transfection (Fig. 3*B*). Considering that the effects of 27-OH on RxRγ, PTBP1 and REST were observed only after 2 DIV, neurons were transfected at 1 DIV. The day after, they were treated with 27-OH (1 μM) and samples for western blot were collected at 3 DIV. The different groups included in the analysis were: transfected with nontargeting siRNA with no treatment (SCR-NT), transfected with nontargeting siRNA and treated with EtOH (SCR-EtOH), transfected with nontargeting siRNA and treated with 27-OH (SCR-27OH), transfected with RxRγ siRNA and no treatment (siRNA-NT), transfected with RxRγ siRNA and treated with EtOH (siRNA-EtOH) and transfected with RxRγ siRNA and treated with 27-OH (siRNA-27OH). As observed in Figure 3*C*,*D*, RxRγ siRNA prevented the 66% increase in RxRγ (SCR-NT = 100 ± 28; SCR-EtOH = 200 ± 46.37; SCR-27OH = 166 ± 46.98; siRNA-NT = 100 ± 46.52; siRNA-EtOH = 101.1 ± 6.77; siRNA-27OH = 86.79 ± 10.31; one-way ANOVA, *P* > 0.05; Fig. 3*C*) and 49% increase in PTBP1 (SCR-NT = 100 ± 28; SCR-EtOH = 173.6 ± 55.27; SCR-27OH = 149 ± 31.29; siRNA-NT = 100 ± 11.49; siRNA-EtOH = 171.4 ± 49.48; siRNA-27OH = 111.2 ± 13.36; one-way ANOVA, *P* > 0.05; Fig. 3*D*) protein levels mediated by 27-OH. We next, evaluated the dendritic arborization of individual neurons transfected at 1 DIV and treated daily with 27-OH (1 μM) until 10 DIV. Neurons were stained with the somatodendritic marker MAP2 and then, the dendritic tree was reconstructed in 3D by confocal microscopy. The morphometric analysis showed that RxRγ silencing at 1 DIV partially prevented the morphological alterations observed in SCR-27OH neurons (Fig. 3*E*). A Sholl analysis showed an increase in dendritic length in siRNA-27-OH neurons (SCR-27OH= 8.56 ± 3.16; siRNA-27OH = 24.26 ± 8.94; two-way ANOVA, *P* < 0.001; *F* = 19.20; Bonferroni post-hoc test, 20, 30 μm, *P* < 0.05; Fig. 3*F*) and in number of intersection (SCR-27OH= 0.96 ± 0.27; siRNA-27OH = 2.57 ± 0.78; two-way ANOVA, *P* < 0.001; *F* = 11.08; Bonferroni post-hoc test, 20 μm, *P* < 0.05; Fig. 3*G*) compared with SCR-27OH neurons. No differences were found between the other treatments regarding the dendritic length neither number of intersections (Fig. 3*F*,*G*; Supplementary Table S1).

### 27-OH Reduces Dendritic Arborization and Spine Density in CA1 Pyramidal Neurons From Cyp27Tg Mice

**Figure 4.**
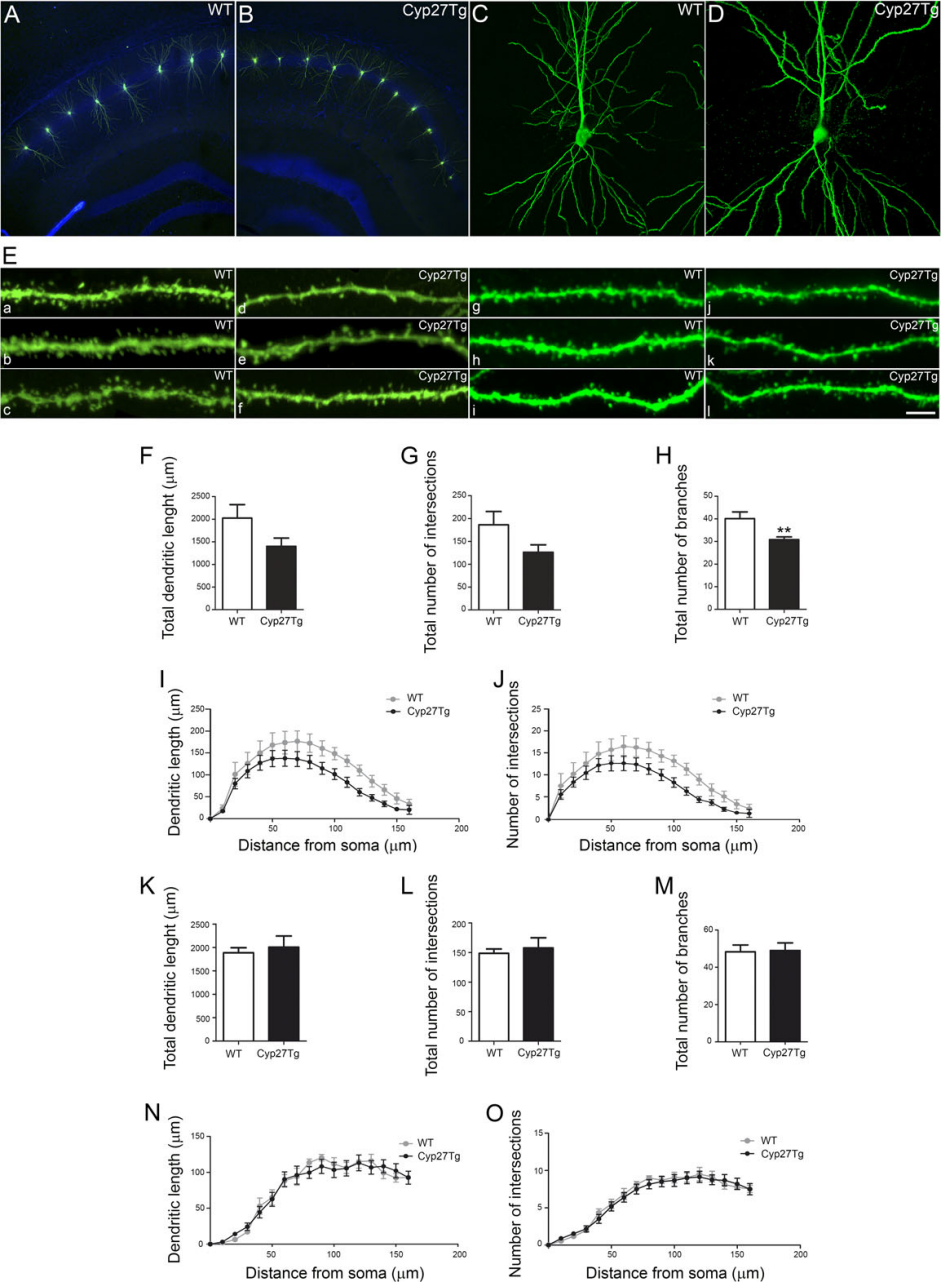
High levels of 27-OH impair neuronal morphology in Cyp27Tg. (*A*, *B*) Confocal images showing Lucifer-yellow (green) injected neurons from hippocampal CA1 region and nuclei (DAPI, blue). (*C*, *D*) Representative pictures showing single injected neurons. (*E*) Representative images of (a–f) individual basal dendrites (*stratum oriens*) from 3 different (a–c) WT mice and (d–f) Cyp27Tg mice. (g–l) Representative images of individual apical dendrites (*stratum radiatum*) from 3 different (g–i) WT mice and (j–l) Cyp27Tg mice. Scale bar (in l): *A*, *B* = 100 μm; *C*, *D* = 50 μm; *E* (a–l) = 10 μm. (*F*–*O*) Morphometric analysis showing (*F*–*H*, *K*–*M*) the average (*F*, *K*) total dendritic length, (*G*, *L*) total number of intersections and (H, M) total number of branches in (*F*–*H*) basal dendritic trees (*stratum oriens*) and (*K*–*M*) apical dendritic trees (*stratum radiatum*). (*I*, *J*, *N*, *O*) Analysis of (*I*, *N*) dendritic length and (*J*, *O*) number of intersections as a function of the distance to the soma in basal dendritic trees (*I*, *J*) and apical dendritic tree (*N*, O). Mann–Whitney test was used to compare averages and two-way ANOVA followed by post-hoc multiple Bonferroni comparison test was used to compare values when presented as a function of the distance from the soma. All data are represented as mean ± SEM; **P < 0.01.

To examine whether high levels of 27-OH could alter dendritic arborization and spine density in vivo we used Cyp27Tg mice. We performed intracellular injections in the hippocampus of 7–8 weeks old WT and Cyp27Tg mice. A total of 182 pyramidal neurons from WT and 178 from Cyp27Tg mice were injected individually with LY in the CA1 region from the hippocampus (Fig. 4). The results were analyzed in 3D by confocal microscopy. The morphological parameters measured were: dendritic length, number of intersections and number of branches (20x/0.75 Dry; pixel size= 0.44, *z*: 1 μm) (Fig. 4*F*–*O*). We evaluated the dendritic arborization of individual neurons from CA1 pyramidal layer in Cyp27Tg mice, both basal dendritic trees (*stratum oriens*; Fig. 4*F*–*J*) and apical dendritic trees (*stratum radiatum*; Fig. 4K-O).

In the *stratum orien*s, for the statistical analysis 6–8 basal branches per animal that were visible were analyzed. We found a decrease of 30% in dendritic length in Cyp27Tg mice compared with WT but not significant statistical differences (WT = 1940 ± 246; *n* = 5; Cyp27Tg = 1398 ± 184; *n* = 5; Mann-Whitney; *P* > 0.05; Fig. 4*F*). The same decrease was found regarding the number of intersections, but no significant statistical differences (WT = 178 ± 24; Cyp27Tg = 126 ± 16; *n* = 5; Mann-Whitney, *P* > 0.05; Fig. 4*G*). However, Cyp27Tg (30.80 ± 1.2; *n* = 5) showed a significant reduction in total number of branches compared with WT mice (39 ± 2.42; *n* = 5; Mann-Whitney, *P* = 0.0079; Fig. 4*H*). No statistical differences were found between WT and Cyp27Tg mice when analyzing dendritic length and number of intersections as a function of the distance from the soma (Sholl analysis; two-way ANOVA, *P* > 0.05; F = 0.45 and 0.41, respectively; Fig. 4*I*, *J*).

In the *stratum radiatum*, for the statistical analysis 5–8 apical branches per animal that were visible were analyzed. No differences were found in Cyp27Tg compared with WT mice in dendritic length (WT = 1887 ± 107; Cyp27Tg = 2005 ± 239; *n* = 7; Mann-Whitney, *P* > 0.05; Fig. 4*K*), number of intersections (WT = 148 ± 7; *n* = 5; Cyp27Tg = 157 ± 17; *n* = 7; Mann-Whitney, *P* > 0.05; Fig. 4*L*) and number of branches (WT = 48 ± 3; *n* = 5; Cyp27Tg = 49 ± 4; *n* = 7, Mann-Whitney, *P* > 0.05; Fig. 4*M*). Neither, in the analysis of dendritic length and number of intersections as a function of the distance from the soma (Sholl analysis; two-way ANOVA, *P* > 0.05; *F* = 0.32 and 0.38, respectively; Fig. 4*N*,*O*).

**Figure 5.**
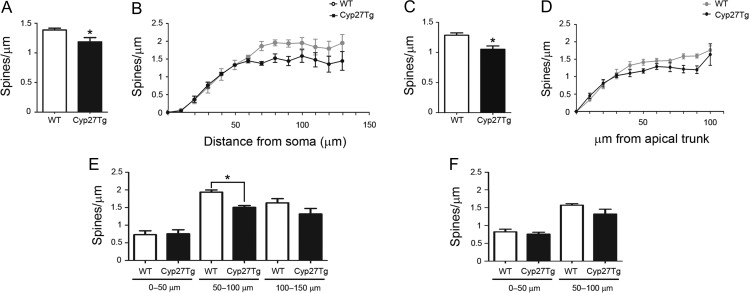
High levels of 27-OH reduce spine density in Cyp27Tg. (*A*–*F*) Comparative morphometric analysis of the spine density (spines/μm) showing (*A*, *C*) the average spine density per dendrite in (*A*) basal dendrites (*stratum oriens*) and in (*C*) apical dendrites (*stratum radiatum*), (*B*, *D*) the spine density as a function of the distance from the soma in (*B*) basal dendrites (*stratum oriens*) and (*D*) apical dendrites (*stratum radiatum*). (*E*, *F*) The average spine density per dendrite as a function of the distance from the soma in (*E*) basal dendrites (*stratum oriens*) (from the soma: 0–50, 50–100,100–150 μm) and in (*F*) apical dendrites (*stratum radiatum*) (from the apical trunk: 0–50, 50–100 μm). Mann–Whitney test was used to compare averages and two-way ANOVA followed by post-hoc multiple Bonferroni comparison test was used to compare values when presented as a function of the distance from the soma. All data are represented as mean ± SEM; *P < 0.05.

A detailed dendritic spine density analysis of the CA1 hippocampal subfield from Cyp27Tg mice was performed. Both, basal dendrites (*stratum oriens*) and apical dendrites (*stratum radiatum*) were included in the analysis. For the statistical analysis 8 dendrites from different neurons per animal were measured (60×/1.4 Oil; pixel size = 0.09, *z*: 0.14 μm; Fig. 5*A*–*F*).

In the *stratum oriens*, spine density was measured assessing a total length of 4990 μm of dendrites in WT and 3569 μm of dendrites in Cyp27Tg mice. As seen in Figure 5*A*, a significant reduction of 19% in the average spine density per dendrite was found in Cyp27Tg (1.12 ± 0.04; *n* = 4; 4302 spines) compared with WT mice (1.38 ± 0.03; *n* = 5; 7114 spines; Mann-Whitney, *P* = 0.0159). No statistical differences were found between WT and Cyp27Tg mice when analyzing spine density as a function of the distance from the soma (Sholl analysis; two-way ANOVA, *P* = 0.17; *F* = 1.4; Fig. 5*B*). However, a further analysis of the average spine density as a function of the distance from the soma (0–50, 50–100, 100–150 μm from the soma; Fig. 5*E*) revealed a significant reduction in the number of spines on the medial portion of the dendrite (50–100 μm; Mann-Whitney, *P* = 0.03).

In the *stratum radiatum*, spine density was examined in apical branches protruding from the main apical trunk. These dendrites were located up to 300 μm from the *stratum pyramidale* (cell body layer). Dendrites located in the *stratum lacunosum-moleculare* were not included in the analysis because due to technical limitations it is very difficult to obtain a sufficient number of labeled neurons reaching this layer as to perform statistical studies. Spine density was measured assessing a total length of 3656 μm of dendrites in WT mice and 3108 μm of dendrites in Cyp27Tg mice. A significant reduction of 18.6% in the average spine density per dendrite was found in Cyp27Tg (1.05 ± 0.05; *n* = 4; 3303 spines) compared with WT mice (1.28 ± 0.03; *n* = 4; 4682 spines; Mann-Whitney, *P* = 0.0286; Fig. 5*C*). No statistical differences were found between WT and Cyp27Tg mice when analyzing spine density as a function of the distance from the soma (Sholl analysis; two-way ANOVA, *P* = 0.48; *F* = 0.97; Bonferroni post-hoc test, *P* > 0.05; Fig. 5*D*) and the average spine density as a function of the distance from the soma (0–50, 50–100 μm from the soma; Mann-Whitney, *P* > 0.05; Fig. 5*F*).

### Cyp27Tg Mice Exhibit Decreased PSD95 Protein Levels and Dysregulation in the REST–miR124a–PTBP1 Axis

**Figure 6.**
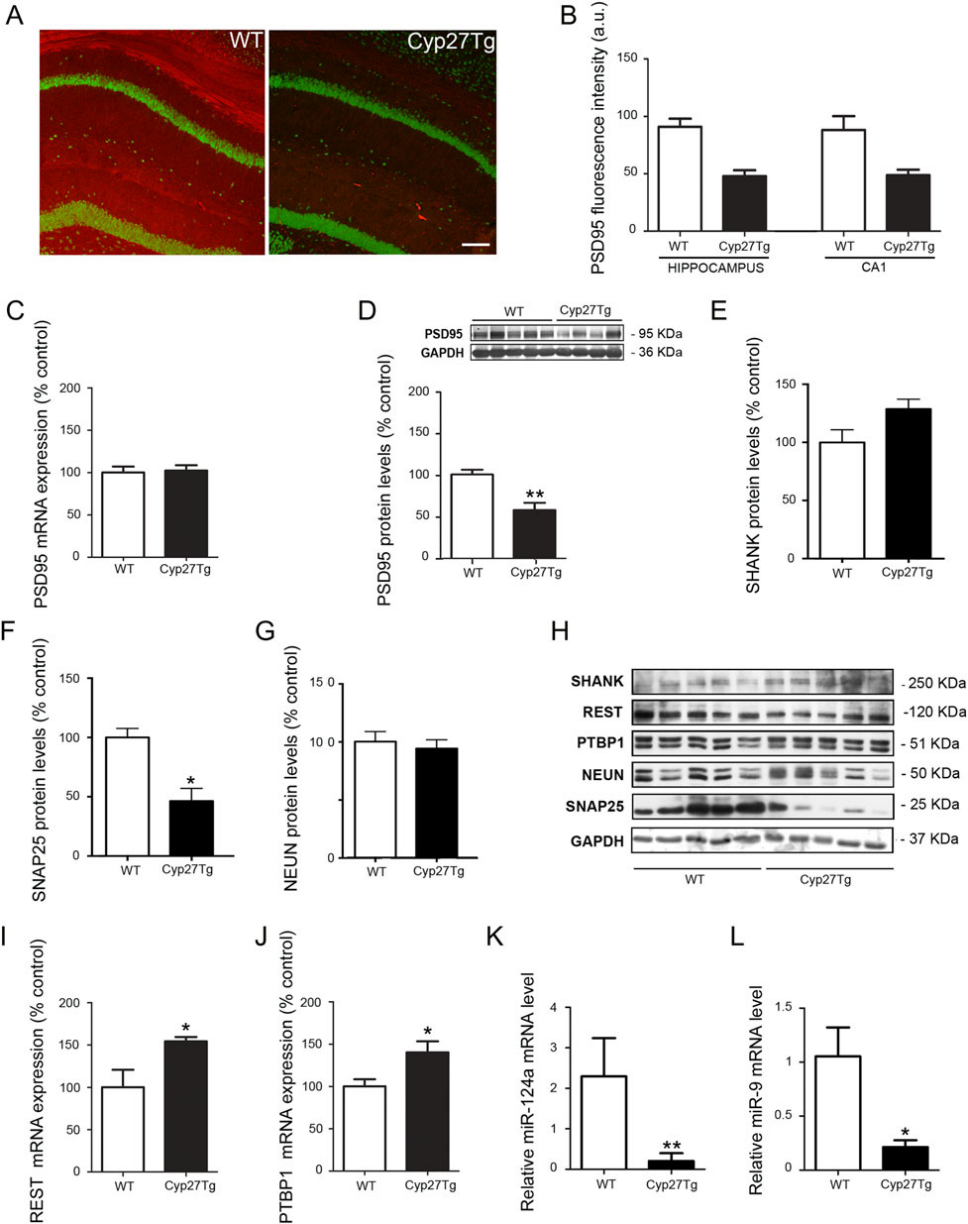
REST-miR124a/9-PTBP1-PSD95 axis dysregulation under high levels of 27-OH in Cyp27Tg. (*A*) Confocal images showing PSD95 (red) and NeuN (green) staining from WT (left panel) and Cyp27Tg (right panel) mice. (*B*) Fluorescence intensity analysis showing the levels of PSD95 in the hippocampus and CA1 from WT and Cyp27Tg mice. (*C*) RT-qPCR measurement and (*D*) western blot analysis and densitometry of PSD95 in Cyp27Tg (*n* = 7) compared with WT mice (*n* = 8). (*H*) Western blot analysis and densitometry of (*E*) SHANK, (*F*) SNAP25 and (*G*) NeuN proteins (*n* = 5). (*I*, *J*) RT-qPCR measurement of (*I*) REST and (J) PTBP1 mRNA expression levels (normalized to GAPDH) for the hippocampus in Cyp27Tg compared with WT mice (*n* = 5). (*K*, *L*) RT-qPCR showing the (*K*) miR-124a and (*L*) miR-9 expression levels in the hippocampus of Cyp27Tg (*n* = 4) compared with WT mice (*n* = 6). Mann–Whitney test was used to compare averages. All data are represented as mean ± SEM; *P < 0.05, **P < 0.01.

PSD95 intensity levels were measured in the CA1 region and the whole hippocampus from Cyp27Tg and WT mice (Fig. 6*A*,*B*). A reduction of 44% in intensity levels was found in Cyp27Tg mice compared with WT in CA1 (WT = 88.15 ± 11.98; Cyp27Tg = 48.83 ± 4.69; *n* = 3; Mann-Whitney, *P* > 0.05), and 52% when the whole hippocampus was measured (WT = 90.99 ± 7.12; Cyp27Tg = 47.96 ± 5.16; *n* = 3; Mann-Whitney, *P* > 0.05; Fig. 6*A*).

Cyp27Tg mice did not differ in levels of PSD95 mRNA in the hippocampus compared with WT mice (102.3 ± 6.39 vs. 100 ± 7.03; *P* = 0.81; *n* = 5) (Fig. 6*C*), but showed a significant reduction of PSD95 protein levels (WT = 100 ± 5.7; *n* = 8; Cyp27Tg = 57.63 ± 8.66; *n* = 7; Mann-Whitney, *P* = 0.022; Fig. 6*D*). Moreover, Cyp27Tg mice showed no differences in SHANK protein levels (WT = 99.92 ± 10.94; Cyp27Tg = 128.7 ± 8.39; *n* = 5; Mann-Whitney, *P* > 0.05; Fig. 6*E*,*H*) and a significant reduction in SNAP25 compared with WT mice (WT = 100 ± 7.53; Cyp27Tg = 46.43 ± 10.68; *n* = 5; Mann-Whitney, *P* = 0.015; Fig. 6*F*,*H*). To attempt a possible neuronal loss in Cyp27Tg mice, NeuN protein levels were measured. No differences were found in Cyp27Tg (93.95 ± 7.51) compared with WT mice (99.95 ± 8.6; *n* = 5; Mann-Whitney, *P* > 0.05; Fig. 6*G*,*H*). Additionally, to elucidate whether the reduction of PSD95 protein levels is accompanied by a dysregulation of the REST–PTBP1 signaling, REST and PTBP1 expression levels were measured. Increased hippocampal REST mRNA expression were found in Cyp27Tg mice compared with WT mice (Cyp27Tg = 154.3 ± 5.22; WT = 100 ± 20.59; *n* = 5; Mann-Whitney, *P* = 0.034; Fig. 6*I*). PTBP1 mRNA expression showed a significant increase in Cyp27Tg mice (140.2 ± 13.11) compared with WT mice (100 ± 8.54; *n* = 5; Mann-Whitney, *P* = 0.033; Fig. 6*J*).

In parallel to the mRNA REST-PTBP1 dysregulation seen in Cyp27Tg mice, we evaluated the levels of miR-124a and miR-9. MiR-124a levels were 92% decreased in Cyp27Tg mice (Cyp27Tg = 0.2053 ± 0.1937; *n* = 4; WT = 2.296 ± 0.9437; *n* = 6; Mann-Whitney, *P* = 0.0048; Fig. 6*K*). MiR-9 levels were also reduced by 80% in Cyp27Tg mice (0.2144 ± 0.06194; *n* = 4) compared with WT mice (1.054 ± 0.2676; *n* = 6; Mann-Whitney, *P* = 0.019; Fig. 6*L*). However, no significant differences were found in REST (WT = 100 ± 14.32; Cyp27Tg = 99.75 ± 5.24; *n* = 5; Mann-Whitney, *P* > 0.05; Fig. 6*H*) and PTBP1 protein levels (WT = 100 ± 10.08; Cyp27Tg = 109.5 ± 6.39; *n* = 5; Mann-Whitney, *P* > 0.05; Fig. 6*H*).

### Intracerebroventricular Injection of 27-OH Increases REST Expression

To validate the notion that high levels of 27-OH play a direct role as a trigger in REST–PTBP1 axis dysregulation, we performed intracerebroventricular (ICV) injections of 27-OH (27CSF) in C57BL/6 J mice. We measured PSD95, REST and PTBP1 hippocampal levels after 24 h of the ICV injection. No significant changes in mRNA expression levels were found in PSD95 (aCSF = 100 ± 5.55; 27CSF = 93.51 ± 2.49; Mann-Whitney, *P* > 0.05; *n* = 5, Supplementary Fig. S3A) and PTBP1 (aCSF, 100 ± 8.20; 27CSF, 98.47 ± 5.82; Mann-Whitney, *P* > 0.05; *n* = 5, Supplementary Fig. S3C) between groups. However, bilateral ICV injections of 27-OH resulted in a significant increase of REST mRNA expression (aCSF = 100 ± 10.68; *n* = 5; 27CSF = 215.3 ± 45.03; *n* = 4; Mann-Whitney, *P* = 0.03; Supplementary Fig. S3B). Additionally, ICV injections of 27-OH did not modify the protein levels of REST (aCSF = 100 ± 10.01; 27CSF = 95.38 ± 8.81; *n* = 5; Mann-Whitney, *P* > 0.05), PTBP1 (aCSF = 100 ± 0.97; 27CSF = 101.5 ± 2.78; *n* = 5; Mann-Whitney, *P* > 0.05) or PSD95 (aCSF = 100 ± 2.57; 27CSF = 101.4 ± 4.44; *n* = 5; Mann-Whitney, *P* > 0.05) (Supplementary Fig. S3D–G).

## Discussion

Hypercholesterolemia in middle age is considered a risk factor for the development of AD and dementia (Kivipelto et al. 2001; Pancani et al. 2013; Knight et al. 2014). In addition hypercholesterolemia in mice is associated with memory deficits (Thirumangalakudi et al. 2008; Heverin et al. 2015). Given the fact that cholesterol itself does not pass the BBB, it is still unclear how high levels of cholesterol in the circulation have an impact on cognition. Accumulating evidence shows that high levels of the BBB permeable cholesterol metabolite, 27-OH, are able to induce neurodegenerative processes leading to cognitive decline (Shobab et al. 2005). Evidences from our laboratory suggest a role for 27-OH as a mediator of neurotoxic effects in the brain in vivo as well as neuronal cells in vitro (Mateos et al. 2011; Ismail et al. 2017). In addition, high plasma levels of 27-OH were recently shown to be a risk factor for cognitive impairment in the elderly (Liu et al. 2016).

In the present study, we found that high levels of 27-OH significantly reduce spine density, dendritic arborization and PSD95 synthesis. Our results point out that decreased PSD95 levels are caused by a REST–miR124a–PTBP1 axis dysregulation and suggest RXRγ as a crucial mediator in the dysregulation of the system.

### 27-OH is a Negative Regulator of Dendritic Arborization

We found that high levels of 27-OH impairs dendritic arborization but do not induce neuronal loss, both in vivo and in vitro. Patterns of dendritic branching may determine the degree to which the integration of inputs is compartmentalized within dendritic arbors. Moreover, the complexity of dendritic structures in large part determines the biophysical properties, which influence their functional capacity (Koch et al. 1982; London and Hausser 2005; van Elburg and van Ooyen 2010). Therefore, our results strongly suggest that high levels of 27-OH reduce the ability of neurons to integrate via inputs, thus inducing neuronal connectivity dysfunction. However, we found a layer-specific significant reduction in the complexity of the dendritic tree from CA1 pyramidal neurons located in *stratum oriens* but no differences in *stratum radiatum* in Cyp27Tg compared with WT mice. These findings suggest that circuits in *stratum radiatum* and *stratum oriens* might be affected differently under high levels of 27-OH.

Furthermore, we found in vivo that high levels of 27-OH produce a significant reduction in miR-124a and miR-9 expression levels. It has been shown that miR-124 induces neurite outgrowth and elongation (Gu et al. 2016). Additionally, miR-9 has been described as a very important factor in proper cortical circuit function, being implicated in neuronal axonal extension and branching. Moreover, miR-9 downregulation has been related with impaired synaptic transmission in the hippocampus (Dajas-Bailador et al. 2012). Therefore, our results show for the first time that high levels of 27-OH impair neuronal architecture and thus, cortical circuits.

### 27-OH Reduces Dendritic Spine Density

Spines on pyramidal neurons are the recipients of most excitatory inputs to the cerebral cortex (DeFelipe and Farinas 1992) and all or almost all dendritic spines establish at least one excitatory glutamatergic synapse (Arellano et al. 2007; Bosch et al. 2015, 2016). Therefore differences in the number of spines probably reflect differences in the number of excitatory inputs that a cell can integrate. We have found that high levels of 27-OH significantly reduce the spine density in hippocampal primary neurons and in CA1 pyramidal neurons from Cyp27Tg, both in apical and basal dendrites. Additionally, in basal dendrites a spine density reduction was found in the medial portion of the dendrites. This anatomic-specific spine density reduction was not found in apical dendrites, suggesting that spines could be affected differently in *stratum oriens* and *stratum radiatum*, and therefore that the circuits in both *strata* might be affected differently when exposed to high levels of 27-OH. Layer-specific morphological alterations on spines in CA1 hippocampal subfield, have been previously described in the transgenic mouse model for AD, APP/PS1 (Merino-Serrais et al. 2011).

Taking into consideration that Cyp27Tg showed a deficit in memory-related test and decreased expression levels of Arc, a memory consolidation marker (Ismail et al. 2017) our findings are consistent with the possibility that high levels of 27-OH produce a synaptic disconnection leading to a cognitive decline.

The present study represents a first step toward the characterization of high levels of 27-OH in dendritic spine density of the CA1 region from the hippocampus, but it would be useful to build upon this by studying additional brain regions that are particularly relevant to memory and cognition. Furthermore, many spines remain apparently normal, suggesting that the reduction in the number of spines might selectively affect certain connections. Indeed, CA pyramidal cells receive inputs from entorhinal cortex, CA3 pyramidal cells, thalamus, amygdala, and neuromodulatory inputs from a number of subcortical nuclei (Andersen et al. 2007). Further studies would be necessary to explore this aspect in more depth.

### 27-OH Induces Synaptic Dysfunction

In this study, we report the novel notion that high levels of 27-OH reduce PSD95 synthesis, both in vivo and in vitro. The postsynaptic protein PSD95 is crucial for the organization of glutamate receptors, adhesion proteins and ion channels (Husi et al. 2000; Fernandez et al. 2009) and for proper synaptic function and maintenance of synaptic connections (El-Husseini et al. 2000). Therefore, inhibition of PSD95 synthesis may impair the function of spines and could be implicated in alterations of the high-order functional connectivity of the cerebral cortex, leading to the cognitive decline that characterizes AD and other neurodegenerative diseases. Additionally, we found that PSD95 intensity levels decreased both, in CA1 and the whole hippocampus under high levels of 27-OH. The results are in accordance with the protein levels measurements but not statistically significant. These findings suggest a general effect of 27-OH in the impairment on hippocampal circuits. Moreover, in Cyp27Tg mice no significant differences were found in the postsynaptic marker SHANK, but a significant decrease was found in the presynaptic marker SNAP25. These results suggest that the postsynaptic dysfunction could be specific for PSD95 whereas the presynaptic structure seems to be impaired under high levels of 27-OH. Further studies should be necessary to explore this result in more depth.

PSD95 synthesis is down regulated by REST, which represses miR-124a, leading to increased PTBP1/PTBP2 ratio. When PTBP1 levels increase, they regulate the splicing of PSD95 to a nonsense-mediated mRNA decay process (Zheng et al. 2012). We show that high levels of 27-OH modulate the expression and protein levels of REST and PTBP1 at early days in vitro, leading to decreased PSD95 mRNA and protein levels. Furthermore, 27-OH treatment for 1–10 DIV leads to the same impairments in dendritic arborization, spine density and PSD95 synthesis as 10 DIV neurons treated only in the first days (1–4 DIV) of neuronal development.

Additionally, our results in vivo show that high levels of 27-OH modulate the mRNA expression levels of REST, PTBP1, miR-124a and miR-9. However, Cyp27Tg did not differ in REST and PTBP1 protein levels compared with WT mice. One explanation could be, that in accordance with the in vitro results, alterations in PTBP1 and REST protein levels occur early in the differentiation process and they might not be present at the studied age in the mouse. However, it is also known that REST regulates its target genes through selective association with other corepressors (Ballas et al. 2005). Thus, its function as a repressor may not necessarily be associated with its protein levels. This is agreement with our findings showing significantly reduced levels of miR-124a and miR-9 found in Cyp27Tg mice. REST is the most well-known repressor of miR-124a expression, thus the decrease found in miR-124a indicates a dysregulation in REST function.

**Figure 7.**
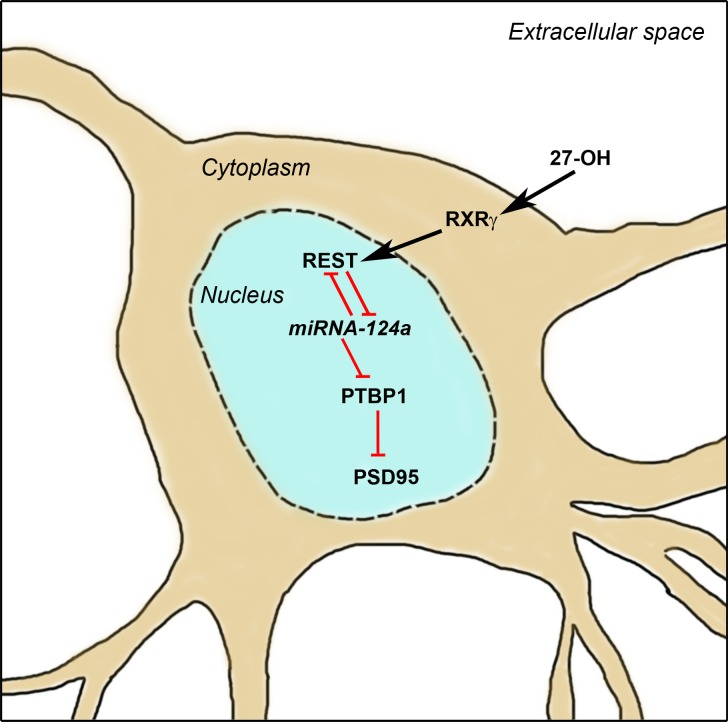
Schematic overview of the proposed mechanism by which 27-OH influences PSD95 synthesis. 27-OH via RXRγ induces a REST–miRNA124a–PTBP1 axis dysregulation leading to a reduction on PSD95 synthesis. Red lines indicate inhibitory pathways.

Together, our in vitro and in vivo results suggest that under high levels of 27-OH, impairments in the REST–miR124a–PTBP1 axis in the beginning of the maturation process are sufficient to trigger morphological alterations and decreased PSD95 levels in differentiated neurons in the hippocampus (Figure 7).

Furthermore, to exclude that the peripheral effects due to high plasma cholesterol levels may produce the effects seen in the hippocampus, we injected a single doses of 27-OH directly into the lateral ventricle of WT mice. We found after ICV injections of 27-OH in mice, changes in REST mRNA expression but not in PTBP1 or PSD95. These results point out 27-OH as a trigger in the REST–miR124a–PTBP1–PSD95 axis dysregulation and suggests that long term exposure to high 27-OH levels at initial stages of the neuronal development are necessary conditions for the 27-OH mediated effects on the PSD95 levels.

Moreover, 27-OH is a well-known endogenous ligand of LXR (Mateos et al. 2011) as well as an endogenous selective ER modulator (Brooks et al. 2017; Raza et al. 2017). We have shown that treatments with LXR ligands do not induce morphological alterations in vitro, or changes in PSD95 levels, which suggests that 27-OH effect is not LXR-dependent. Treatments with β-Estradiol, ICI, and 27-OH show that 27-OH induces its effect on dendritic arborization and PSD95 levels independently of the presence of β-Estradiol and ICI, and thus, it is not a mechanism mediated by ER.

Therefore, we hypothesized that the adverse effects of 27-OH on neuronal morphology and PSD95 synthesis might be mediated by alternative receptors to LXR and ER. All 4 isoforms of RxRs exist in neuronal cells; however, RxRγ has been implicated in the control of cholesterol metabolism (Shulman and Mangelsdorf 2005), neuronal differentiation (Diez del Corral et al. 2003) and neurite growth (Calderon and Kim 2007). Moreover, several evidence indicate that activated RxRγ can translocate to the nucleus and promote transcription of genes regulating neuronal specification including REST (Singh et al. 2011).

Our results showed an increase in RxRγ mRNA expression levels in neurons from primary culture after 2DIV. RxRγ knockdown with a selective siRNA prevented the increase in PTBP1 protein levels and partially recovered the morphological alterations observed in neurons treated with 27-OH. These results suggest that RxRγ could be an important mediator in the 27-OH effect on PTBP1 upregulation, pointing out a crucial role for RxRγ in the REST–miR124a–PTBP1–PSD95 axis dysregulation and neuronal morphology in the brain. However, in the siRNA transfection experiments, we observed an increase in RxRγ and in PTBP1 protein levels in silenced neurons treated with Et-OH. We found that Et-OH increases PTBP1 protein levels by an RxRγ independent mechanism. Thus, taking into account that siRNA experiments were not statistically significant, we conclude that further experiments are needed to confirm the specific role of RxRγ in the REST–miR124a–PTBP1–PSD95 axis dysregulation.

In favor of our hypothesis, the morphological analysis showed that the neurons treated with Et-OH did not differ from Et-OH untreated neurons indicating that, the effect of 27-OH on neuronal morphology is not Et-OH dependent.

It is known that altering the amount of cholesterol causes loss of spines and synapses postsynaptically (Sooksawate and Simmonds 2001). Taking into consideration that 27-OH has been proposed as a physiological inhibitor of cholesterol synthesis in the brain (Sever et al. 2003; Goldstein et al. 2006; Radhakrishnan et al. 2007; Ali et al. 2013), high levels of 27-OH could be expected to modulate cholesterol metabolism and interfere with neurotransmission.

The role of hypercholesterolemia as a risk factor in the pathogenesis of dementia is not fully understood. High blood cholesterol levels itself, though correlating with the risk of develop neurodegeneration, have not revealed any links to causation or mechanism (Reitz 2012). Thus, new targets may lead the way to alternative therapeutic strategies hindering the progression of AD and other neurodegenerative diseases. It is well documented that aberrant CSF-levels of 27-OH are found in patients with mild cognitive impairment (Leoni and Caccia 2011; Mateos et al. 2011) and that 27-OH accumulates in the brain of AD patients (Heverin et al. 2004). Furthermore, 27-OH levels correlate with cholesterol levels in the blood (Babiker et al. 2005).

Therefore, reducing the overproduction of 27-OH using inhibitors for the enzyme responsible for converting cholesterol to 27-OH, Cyp27A1, may be a possible preventive strategy to reduce the risk of dementia or to improve therapies restoring neuronal function in neurodegeneration. Indeed, in the periphery, Cyp27A1 as a drug target to reduce the levels of 27-OH has been featured revealing that 27-OH can be decreased without altering cholesterol metabolism (McEwen and Reagan 2004; Mast et al. 2015). Additionally, it has been shown that a reduction of 27-OH production in hypercholesterolemic rats using an inhibitor of Cyp27A1, resulted in improved memory function (Zhang et al. 2018).

To summarize, our findings indicate that 27-OH is an important mediator between hypercholesterolemia and neuronal and synaptic dysfunction. In combination with our previous results the present findings are clearly consistent with 27-OH as a possible target for a drug discovery program

## Supplementary Material

Supplementary Data
