# Supplementary material for: 27-Hydroxycholesterol Induces Aberrant Morphology and Synaptic Dysfunction in Hippocampal Neurons
Source: Cereb Cortex. 2018 Nov 3;29(1):429–46. doi: 10.1093/cercor/bhy274 (PMC6294414; doi:10.1093/cercor/bhy274)
Supplement: Supplementary Data [file bhy274_supplementarymaterial.pdf]

Supplementary material

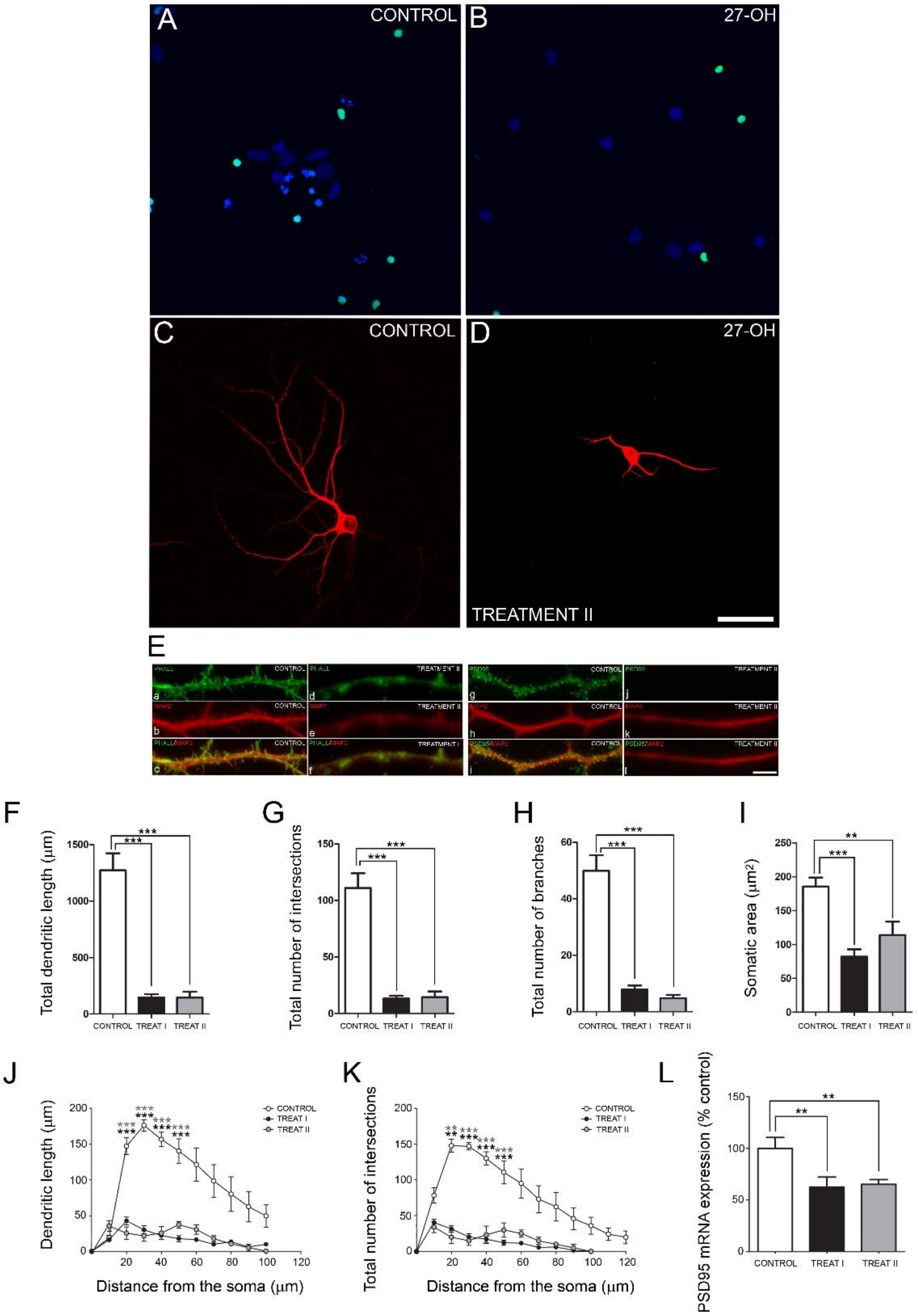

Supplementary Figure 1. 27-OH treatments modulates neuronal arborization, spine density, PSD95 synthesis and the REST-PTBP1-PSD95 axis *in vitro*

(A, B) Confocal images showing TUNEL ASSAY performed in (A) control neurons and (B) neurons treated with 27-OH. In DAPI are stained all neurons and in green colour the apoptotic neurons. (C, D) Confocal images showing the morphological structure of (C) control neurons and (D) neurons under treatment II with the somatodendritic marker MAP2. (E) Representative images of dendrites from control neurons (a-c, g-i) and treatment II (d-f, j-l) stained using phalloidin (PHALL) (a,c,g,i), anti-MAP2 (b,c,e,f,h,i,k,l) and anti-PSD95 (g-i,f,l). Neurons treated with 27-OH under treatment II, showed less dendritic spines and PSD95 compared to control neurons. (F-L) Comparative morphometric analysis of (F, J) dendritic length, (G, K) number of intersections, (H) number of branches and (I) somatic area for control neurons and neurons under treatment II (n= 6). Data presented as (F-I) the average per neuron and (J, K) as a function of distance from the soma (n= 6). (F-I) One-way ANOVA test followed by a *post-hoc* multiple Bonferroni comparison test was used to compare averages, (J, K) two-way ANOVA followed by a *post-hoc* multiple Bonferroni comparison test was used in sholl analysis. (L) 27-OH treatments (Treatment I and Treatment II) modulate PSD95 expression levels *in vitro*. One-way ANOVA test followed by a *post-hoc* multiple Bonferroni comparison test was performed. Data from Figure 1 for 27-OH treatment (treatment I) was included in the graph for a better exhibition of the results. Scale bars (in F) A-C, (in r) G: 10µm. All data are represented as mean ± SEM; ** $P < 0.01$, *** $P < 0.001$.

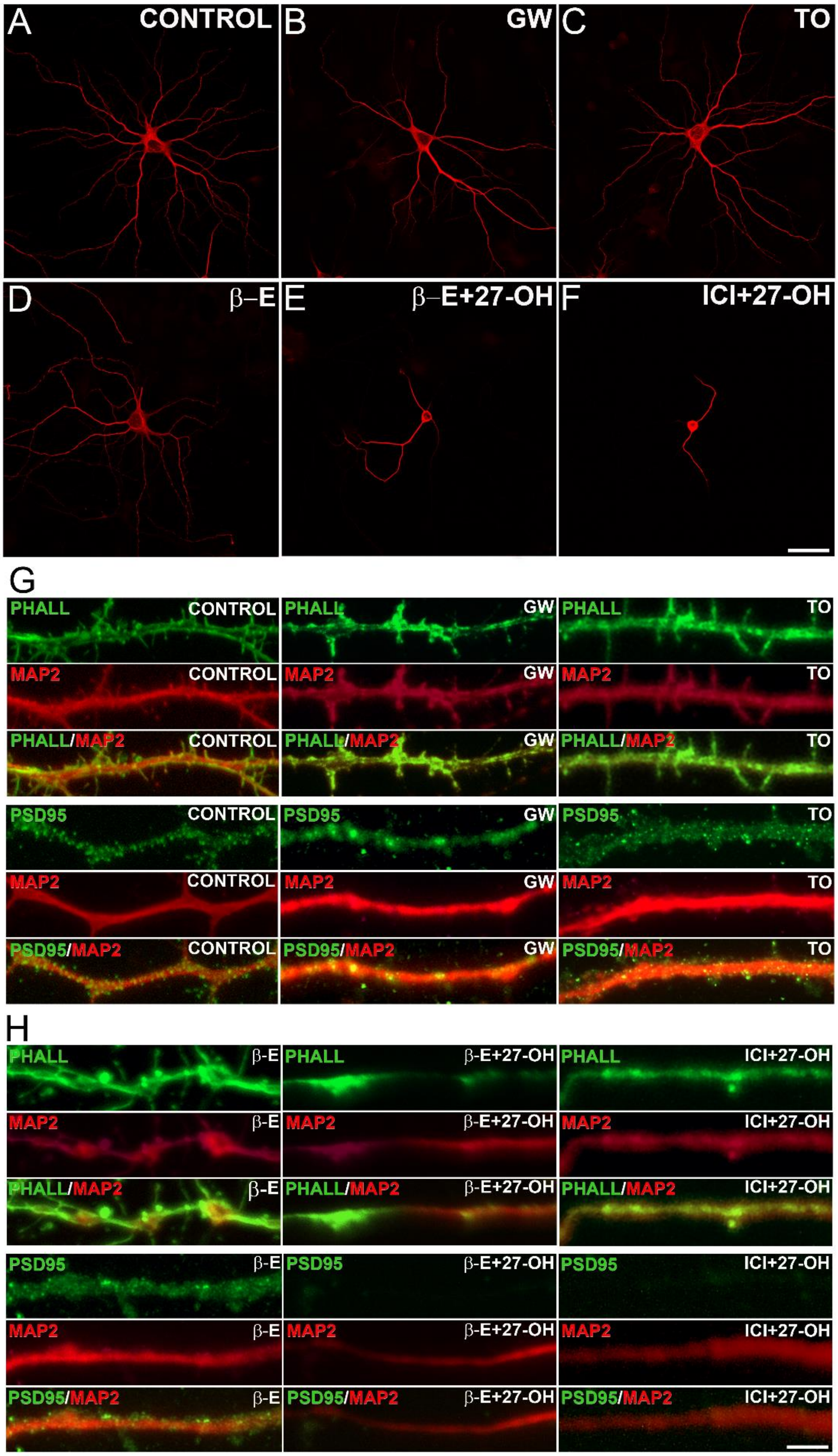

Supplementary Figure 2. Role of LXR and ER on 27-OH treatment *in vitro*.

Higher magnification from Figure 2. (A-F) Confocal images showing the dendritic arborization using the somatodendritic marker MAP2 of (A) control neurons, neurons treated with the LXR ligands: (B) GW3965 (GW, 1 μM) and (C) TO-901317 (TO, 1 μM), (D) β-Estradiol (β-E; 10 nM), (E) β-Estradiol +27-OH (β-E+27-OH; 10 nM and 1 μM respectively) and (F) the ERs antagonist + 27-OH (ICI+27-OH; 100 nM and 1 μM respectively). (G, H) Representative images of dendrites from control neurons and neurons treated with (G) GW, TO; (H) β-E, β-E+27-OH and β-E+ICI stained using phalloidin (PHALL), anti-MAP2 and anti-PSD95. Scale bar (in F): A-F: 30µm; G, H: 10µm.

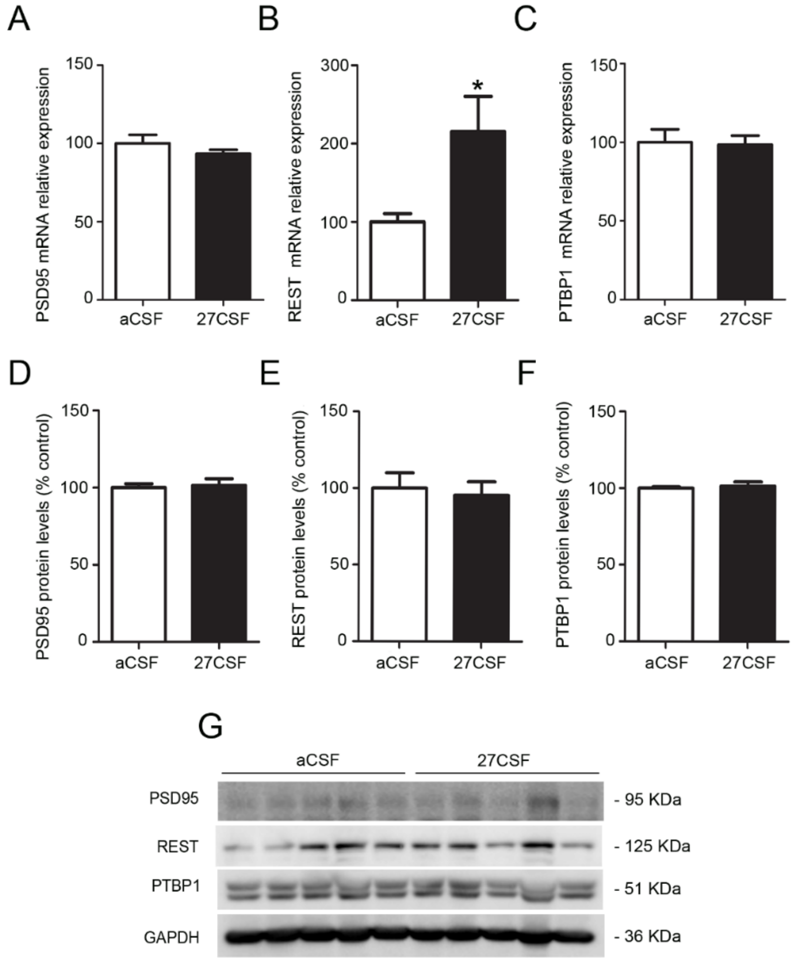

Supplementary Figure 3. Upregulation of mRNA expression of REST after 27-OH intracerebroventricular injections *in vivo*.

24 hours later to the intracerebroventricular (ICV) injections of CSF (aCSF) and 27-OH (27CSF) (5 μM), the hippocampi of the mice were analyzed (A-C) by Real-time RT-PCR and (D-G) by Western blot for PSD95, REST and PTBP1 (normalized to GAPDH) (n = 5). (B) REST mRNA expression showed a significate increase in 27CSF compared with aCSF. (A, C) No differences were found in the mRNA analysis of PSD95 and PTBP1. (D-G) Western blot showing no significantly differences between groups. (G) Representative immunoblot of aCSF and 27CSF showing PSD95, REST, PTBP1 and GAPDH. Mann-Whitney test was employed. All data are represented as mean ± SEM;* $P < 0.05$.

		SCR-NT	SCR-ETOH	SCR-27-OH	siRNA-NT	siRNA-ETOH	siRNA-27OH
**Dendritic length**	**SCR-27OH**	20,30,<br>40=***	20,30,<br>40=***		20,30,<br>40=***	20,30,<br>40=***	20=*
	**siRNA-27-OH**	20,30,<br>40=***	20,30,<br>40=***	20,30=*	20,30,<br>40=***	20,30,<br>40=***	
**Number of Intersec.**	**SCR-27OH**	10=**<br>20,30,<br>40=***	10=*<br>20,30,<br>40=***		10,20,30,<br>40=***	10,20,30,<br>40=***	20=*
	**siRNA-27-OH**	20,30,<br>40=***	20,30,<br>40=***	20=*	10=**<br>20,30,<br>40=***	10,20,30,<br>40=***	

Supplementary Table 1. Morphometric analysis in function of the distance from the soma (Sholl analysis). Comparative morphometric analysis of dendritic length and number of intersections in function of the distance from the soma between groups (20, 30 and 40 μm from the soma). The groups included in the analysis were: neurons transfected with non-targeting siRNA with no treatment (SCR-NT), transfected with non-targeting siRNA and treated with EtOH (SCR-EtOH), transfected with non-targeting siRNA and treated with 27-OH (SCR-27OH), transfected with RxRγ siRNA and no treatment (siRNA-NT), transfected with RxRγ siRNA and treated with EtOH (siRNA-EtOH) and transfected with RxRγ siRNA and treated with 27-OH (siRNA-27OH). Data are presented as significant values (Two-way ANOVA, $P<0.001$) between groups in a *post-hoc* multiple Bonferroni comparison test. All data are represented as mean ± SEM; * $P < 0.05$, ** $P < 0.01$, *** $P < 0.001$.
